# Novel heterozygous *BPIFC* variant in a Chinese pedigree with hereditary trichilemmal cysts

**DOI:** 10.1002/mgg3.697

**Published:** 2019-04-29

**Authors:** Xian‐Guo Fu, Zhao Huang, Su‐Juan Zhou, Jing Yang, Yun‐Juan Peng, Luo‐Yuan Cao, Hua Guo, Guang‐Hui Wu, Ying‐Hua Lin, Bao‐Ying Huang

**Affiliations:** ^1^ Department of Central Laboratory Ningde Municipal Hospital, Fujian Medical University Ningde Fujian China; ^2^ Department of Clinical Laboratory Ningde Municipal Hospital, Fujian Medical University Ningde Fujian China; ^3^ Department of Pathology Ningde Municipal Hospital, Fujian Medical University Ningde Fujian China; ^4^ Department of Neurosurgery Ningde Municipal Hospital, Fujian Medical University Ningde Fujian China

**Keywords:** hereditary trichilemmal cysts, *BPIFC*, whole‐exome sequencing

## Abstract

**Background:**

Trichilemmal cysts (TCs) are common intradermal or subcutaneous cysts, which are commonly sporadic and rarely autosomal dominantly inherited. However, little is known about the disease‐determining genes in families with TCs exhibiting Mendelian inheritance.

**Objective:**

The aim of this study was to identify the causative gene in a family with TCs.

**Methods:**

Whole‐exome sequencing was performed on a TCs family to identify the candidate gene. Sanger sequencing was conducted to validate the candidate variants and familial segregation.

**Results:**

We identified the heterozygous variant c.3G>C (p.Met1?) within the *BPIFC* gene. Sanger sequencing confirmed the cosegregation of this variant with the TCs phenotype in the family by demonstrating the presence of the heterozygous variant in all the 12 affected and absence in all the seven unaffected individuals. This variant was found to be absent in dbSNP141, 1,000 Genomes database and 500 ethnicity matched controls.

**Conclusion:**

Our results imply that *BPIFC* is a causative gene in this Chinese family with hereditary TCs. Further studies should be performed to validate the role of *BPIFC* in the pathogenesis of this disease.

## 1 INTRODUCTION

Trichilemmal cysts (TCs), also known as pilar cysts, are common intradermal or subcutaneous cysts, occurring in 5%–10% of the population with a female predominance incidence (Eiberg et al., 2005; Goldsmith, Katz, Gilchrest, & Paller, 2012). TCs are predominantly on the scalp, and are keratin‐filled, epithelial‐lined cysts arising from outer root sheath of the hair follicle (Goldsmith et al., 2012; McGavran & Binnington, 1966; Pinkus, 1969). These cysts are lined by stratified squamous epithelium undergoing trichilemmal keratinization and resulting in a cyst wall without a granular layer (Goldsmith et al., 2012). They are almost always benign, rarely lead to proliferating TCs and very rarely malignant transformation (Brownstein & Arluk, 1981; Weiss, Heine, Grimmel, & Jung, 1995).

TCs are commonly sporadic and rarely autosomal dominantly inherited. The first description of autosomal dominant inheritance of TCs was reported by Leppard and Sanderson (1976) and Leppard, Sanderson, and Wells (1977). A panel of clinical and histologic criteria for identifying the hereditary TCs has been proposed (Seidenari et al., 2013), which includes: the diagnosis of TCs in at least two first‐degree relatives, or in three first‐ or second‐degree relatives in two consecutive generations; TCs diagnosed at less than 45 years of age in at least one family member; and the diagnosis of multiple cysts or giant cysts (greater than 5 cm) or cysts with rare histopathologic features, such as proliferation or ossification. In hereditary cases, there was a younger age of onset and greater presence of multiple cysts compared to sporadic cases. So identifying the traits of hereditary TCs has important implications. Several genetic studies regarding hereditary TCs have previously been reported. By linkage analysis in a Danish family segregating TCs, a 10.3‐Mb candidate disease locus, termed TRICY1 (OMIM# 609649), on chromosome 3p24‐p21.2 was identified, but sequencing analysis failed to detect variants in two candidate genes in this region previously reported in inherited hair defects: *CTNNB1* and *MLH1* (Eiberg et al., 2005). Likewise, the *PTCH1* gene is confirmed to be unrelated to the disease (Seidenari et al., 2013). Therefore, the disease‐determining gene of hereditary TCs remains unknown.

In this study, we identified a heterozygous variant within the *BPIFC* (OMIM# 614109) gene that cosegregated with the phenotypes of a Chinese TCs family with an autosomal‐dominant inheritance using whole‐exome sequencing (WES).

## 2 MATERIALS AND METHODS

### 2.1 Ethical compliance

The protocols of this study were evaluated and approved by the Ethics Committee of Ningde Municipal Hospital of Fujian Medical University.

### 2.2 Subjects

A TCs family from the Chinese Han population participated in this study. The proband was examined with multiple mobile, firm, well‐circumscribed nodules over their scalp. The nodules were excised and diagnosed by histopathological examination (HPE). Peripheral blood from this family members were collected after signing informed consent. In addition, 500 ethnicity‐matched subjects were enrolled as controls.

### 2.3 WES and analysis

The genomic DNA was extracted from peripheral blood white cells by using the QIAamp DNA blood kit (Qiagen, Germany). Two probands (III1, III2) were selected for WES.

Library preparation and a sequencing run were performed using SureSelect Human All Exome enrichment kit (Agilent) and Illumina Hiseq2000 platform (Illumina). Raw sequencing reads were subsequently mapped to the human reference genome (GRCh37/HG19) using the alignment tool Burrows‐Wheeler Aligner (BWA V0.7.12) (Li & Durbin, 2009). Variant calling and local realignment around insertions or deletions (Indels) analyses were performed using the Genome Analysis Tool Kit (DePristo et al., 2011; Van der Auwera et al., 2013), annotated by ANNOVAR(Wang, Li, & Hakonarson, 2010). Based on functional annotations, variants were filtered first for the nonsynonymous mutations (NS), splice acceptor and donor site variants (SS), and coding indels, and then filtered comparing to the dbSNP database (V.141). Variants with minor allele frequency ≥1% according to allele frequency from publicly available databases such as 1,000 Genomes Project were filtered and excluded. Furthermore, in silico tools such as SIFT, PolyPhen2 and MutationTaster were used to predict the pathogenicity of the variant (Adzhubei et al., 2010; Kumar, Henikoff, & Ng, 2009; Schwarz, Cooper, Schuelke, & Seelow, 2014). The variants were shared by two probands but not presented in the public databases were considered to be the candidate variants.

### 2.4 Sanger sequencing

The candidate variants identified from WES were confirmed in the affected individuals from this TCs family using Sanger sequencing; segregation patterns were obtained to determine whether the variant cosegregated with the TCs phenotype in the pedigree. Then, the confirmed candidate variant was screened in 500 unrelated individuals of matching Han Chinese ancestry to evaluate its frequency in the general population. PCR primers (Table S1) were designed using the Primer3 web program (version 4.1.0).

### 2.5 Genetic linkage analysis

Linkage analyses were performed using MERLIN software (Abecasis, Cherny, Cookson, & Cardon, 2002). An autosomal dominant model was assumed, and the disease allele frequency was set at 0.0001, the penetrance of carriers and noncarriers was set at 0.99 and 0, respectively.

## 3 RESULTS

### 3.1 Clinical characterisations

**Figure 1:**
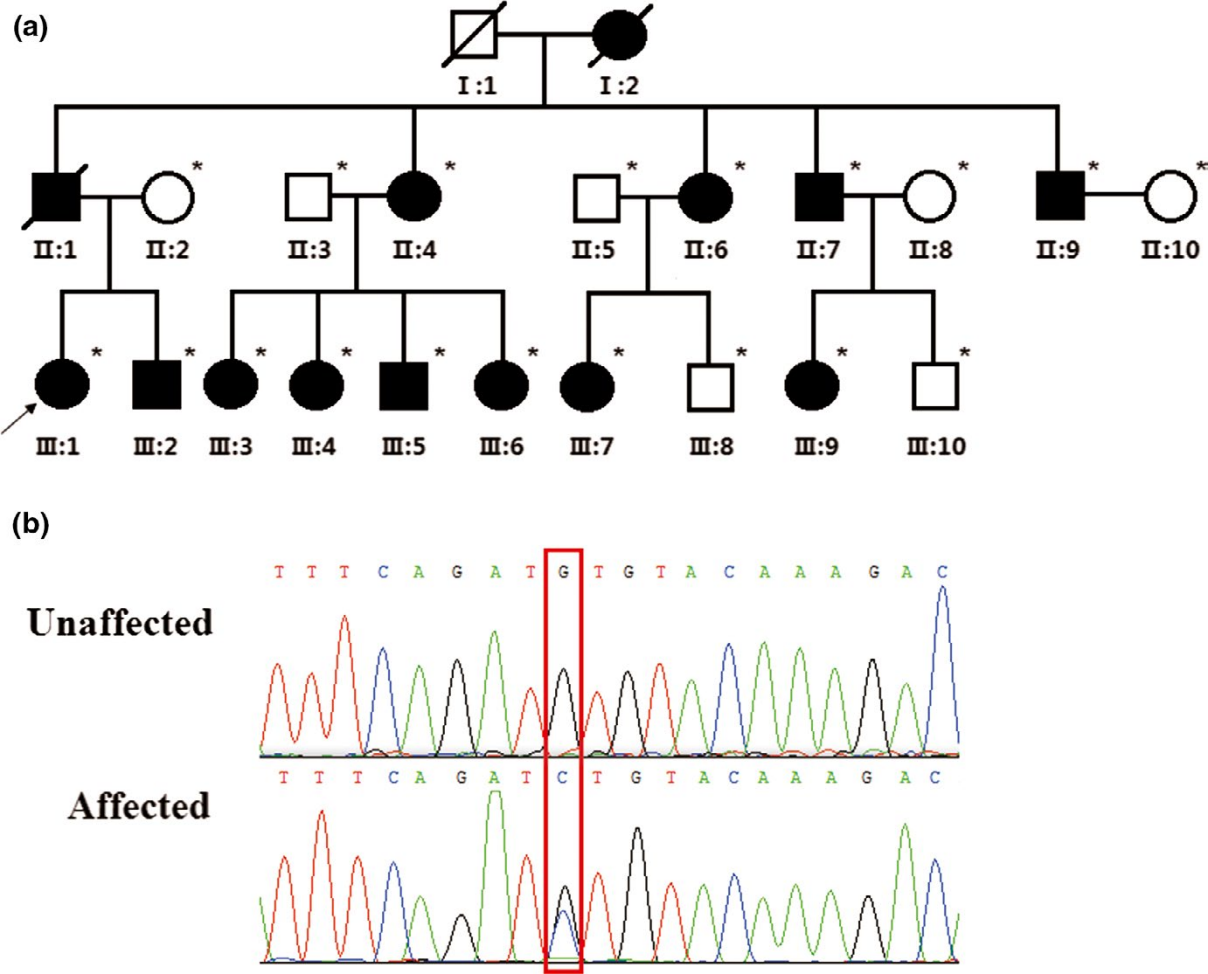
The *BPIFC* gene c.3G＞3 variant identified in the hereditary trichilemmal cysts (TCs) family. (a) Pedigree and segregation pattern in the hereditary TCs family (asterisks indicate genomic DNA available in this study). (b) Chromatogram of the heterozygous c.3G>A variant

**Figure 2:**
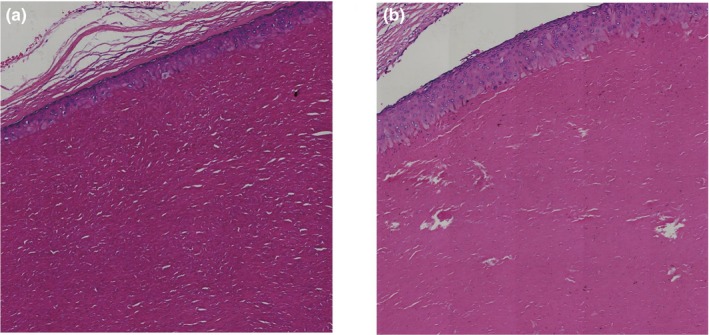
Trichilemmal cysts diagnosed by histopathological examination (HPE) from the affected individuals in this hereditary TCs family (III1 (a) and III3 (b)). The cysts showing an epithelial lining that lacks a granular layer

A three‐generation Han Chinese family that includes 12 individuals exhibited an autosomal dominant mode of inheritance of TCs (Figure 1). Notably, the female members were predominantly affected and presented with a more severe phenotype than males. The proband in our study (III1) is a 36 years old female presented with multiple mobile, firm, well‐circumscribed nodules over their scalp, who underwent an excision of multiple TCs that diagnosed by HPE in 2010 (Figure 2a). In another affected female (III3), three excised cysts over the scalp were also diagnosed with TCs by HPE (Figure 2b). After taking a careful family history, eight individuals (I2, II1, II4, II6, III1, III2, III3, III5) in this pedigree had similar cysts predominantly over the scalp, and six individuals (II7, II9, III4, III6, III7, III9) had cysts occasionally over the face, chest or arm. These features confirmed the hereditary form of TCs in this family, according to the diagnosis criteria of hereditary TCs (Seidenari et al., 2013).

### 3.2 A heterozygous variant was identified in *BPIFC*

An average of 7.28 billion bases of high‐quality sequence was generated by WES, with 54.25% of the total bases mapped to the reference genome with a mean coverage of 63×. On average per sequencing individual, 96.75% of the exome were covered at least 10×. Finally, a total of 11,797 genetic variations, including nonsynonymous mutations (NS), splice acceptor and donor site variants (SS), and coding insertions or deletions (Indels), were identified in two affected individuals. These variants were then filtered using two criteria: if a variant was predicted to have functional impact by computational predictive programs, such as SIFT, PolyPhen2, MutationTaster; and if it was found in two affected individuals and absent from the dbSNP141 and 1,000 Genomes database.

After variants filtering, we identified 52 novel NS/SS/Indels that were shared by two individuals in this family (Table S2). We focused on the two heterozygous variants: a nonsynonymous variant in *DLEC1* (3p21.3) and a nonsynonymous variant in *BPIFC* (22q12.3). We first used Sanger sequencing to confirm the occurrence of these genetic variants in all the available affected and unaffected individuals of this family. We could not detect the heterozygous variant in *DLEC1* in the other affected individuals, although the variant was locating in the predicted hereditary TCs linkage regions on 3p24‐p21.2 established in previous study (Eiberg et al., 2005). Fortunately, Sanger sequencing demonstrated that all the 12 affected individuals carried the heterozygous variant in *BPIFC* (NM_174932.2: c.3G>C: p.Met1?) which was absent in the seven unaffected individuals of this family (Figure 1b). Genetic linkage analysis was performed to assess the statistical significance of the observed cosegregation between the heterozygous variant and hereditary TCs in the Chinese family. An autosomal dominant model was assumed, in which the disease allele frequency was set at 0.0001, and the penetrance of carriers and noncarriers was set at 0.99 and 0, respectively. The logarithm of odds (LOD) score was 3.114 in this family, suggesting that the observed cosegregation is unlikely to have occurred by chance.

Furthermore, this variant was absent in the dbSNP141 and 1,000 Genomes databases and not detected in the 500 population‐matched normal controls. In silico analyses about the potential effect of c.3G>C on *BPIFC* function shown that this variant was predicted to be “Deleterious,” “Probably Damaging,” and “Disease causing” by SIFT, Polyphen2, and Mutation Taster, respectively. All these results suggested that this variant is a causal variant for hereditary TCs, instead of a rare polymorphism.

## 4 DISCUSSION

In this study, we identified a heterozygous variant in *BPIFC* (NM_174932.2: c.3G>C: p.Met1?) that segregated with the phenotypes in the family. This variant was absent from the dbSNP141 and 1,000 genomes database and 500 population‐matched normal controls. Taken together, our findings have strongly implicated *BPIFC* as a candidate causative gene for hereditary TCs.

The *BPIFC* gene (MIM# 614109) was identified from a fetal skin cDNA library and located on chromosome 22q12.3 in 2002, which spans 15 exons and 14 introns (Mulero et al., 2002). The gene encodes bactericidal/permeability‐increasing protein fold–containing family member C protein (BPIFC), also known as bactericidal/permeability‐increasing protein like 2 (BPIL2), belongs to the BPI fold‐containing (BPIF) superfamily. The BPIF superfamily comprises a heterogeneous group of 13 proteins including palate, lung, and nasal epithelium clone (PLUNC) family proteins, BPI protein, lipopolysaccharide‐binding protein (LBP), phospholipid transfer protein (PLTP), cholesterol ester transfer protein (CETP), and BPIFC (Bingle, Seal, & Craven, 2011; Bingle & Bingle, 2011; Masson, Jiang, Lagrost, & Tall, 2009). These proteins share highly similar secondary structure with the BPI fold, which consists of a boomerang shaped structure composed of two distinct N‐ and C‐terminal domains (Beamer, Carroll, & Eisenberg, 1998). The BPIF family mainly involves in the innate immune system and lipoprotein metabolism. BPI and LBP play an important role in the host defense against gram‐negative bacteria (GNB) infection and endotoxin‐induced inflammation owing to a high affinity in its N‐terminal domain for the lipopolysaccharides of GNB (Gazzano‐Santoro et al., 1992; Schultz & Weiss, 2007). PLUNC proteins are predominantly expressed in the upper airways and have host‐protective and immunomodulatory functions in response to inflammation (Britto & Cohn, 2015; Sayeed, Nistico, St Croix, & Di, 2013). CETP and PLTP are human plasma proteins and both play a major role in lipoprotein metabolism (Chowaniec & Skoczynska, 2018). However, less is known about the functionality of BPIFC protein. BPIFC protein is mainly expressed in human epidermis (Toulza et al., 2007) and highly expressed in the basal layer of the epidermis from inflammatory skin of patients with psoriasis (Mulero et al., 2002). In zebrafish, BPIF family C, like (*bpifcl*) modulates the expression of Kisspeptin that a molecule known to be affected by inflammation in the brain (Moriya, Tahsin, & Parhar, 2017). Comparison of the mouse and human orthologs showed that BPIFC protein is evolving rapidly, having an elevated Ka/Ks ratio similar to the PLUNC protein family and BPI protein (Bingle et al., 2004), which implies that BPIFC protein is involved in host defense. Taken together, these data suggest that the BPIFC protein may play a role in inflammation and innate immunological functions in human epidermis, just like the other members of BPI‐fold family. Furthermore, in the PLUNC protein family, SPLUNC1 and LPLUNC1, which are highly expressed in nasopharyngeal epithelium, play a significant role in the process of chronic inflammation and carcinogenesis of nasopharyngeal epithelium by suppressing IL‐6‐induced nasopharyngeal carcinoma cell proliferation through activating NF‐κB and STAT3 signaling pathways (Liao et al., 2014) or down‐regulating of the MAP kinase and cyclin D1/E2F pathways (Yang et al., 2013). Does it imply that the BPIFC protein, like the PLUNC family proteins, plays a role in the inflammation and then the formation of cyst, which remains to be validated by further studies.

In conclusion, our study first identified *BPIFC* as a disease gene in the three‐generation hereditary TCs family of Chinese population by combining WES with segregation analysis. Further functional studies and animal studies of the *BPIFC* gene would shed new insights into the genetic etiopathogenesis of hereditary TCs.

## CONFLICT OF INTEREST

The authors declare that they have no conflict of interest.
