# Supplementary material for: Novel heterozygous BPIFC variant in a Chinese pedigree with hereditary trichilemmal cysts
Source: Mol Genet Genomic Med. 2019 Apr 29;7(6):e697. doi: 10.1002/mgg3.697 (PMC6565563; doi:10.1002/mgg3.697)
Supplement: Supplementary file 1 [file MGG3-7-e697-s001.docx]

Supp. Table S1 Primers for candidate genes

| Gene | OMIM | Genebank | Forward primer | Reverse primer |
| --- | --- | --- | --- | --- |
| DLEC1 | *604050 | NC_000003.12 | GGACCTGCTGTGTGAAATCG | ACACACCAGGGCAAGTTTCA |
| BPIFC | *614109 | NC_000022.11 | TGGATGGATGTATTGAGTTCTGG | TCGCAGCCATCTTAGCAGTT |
