# Supplementary material for: Novel heterozygous BPIFC variant in a Chinese pedigree with hereditary trichilemmal cysts
Source: Mol Genet Genomic Med. 2019 Apr 29;7(6):e697. doi: 10.1002/mgg3.697 (PMC6565563; doi:10.1002/mgg3.697)
Supplement: Supplementary file 2 [file MGG3-7-e697-s002.doc]

**Supp. Table 2 Candidate variants after the WES data filtering**

| Chr | Gene | Func | Transcript |
| --- | --- | --- | --- |
| chr1 | NUAK2 | missense_variant | NM_030952.1:p.Arg569Gln/c.1706G>A |
| chr2 | ITGB6 | missense_variant | NM_000888.4:p.Phe390Leu/c.1168T>C |
| chr2 | ANKRD36 | frameshift_variant | NM_001164315.1:p.Ala395fs/c.1183_1184delGC |
| chr2 | ANKRD36 | frameshift_variant | NM_001164315.1:p.Val396_Asp397fs/c.1187_1188insTT |
| chr3 | ZNF717 | frameshift_variant | NM_001290210.1:p.Glu113fs/c.337delG |
| chr3 | ARL13B | splice_donor_variant | NR_033427.1:n.527_528insT |
| chr3 | DLEC1 | stop_gained | NM_007337.2:p.Gln888*/c.2662C>T |
| chr4 | SLC9B1 | frameshift_variant | NM_139173.3:p.Val446fs/c.1338_1339delGT |
| chr6 | RSPH3 | missense_variant | NM_031924.4:p.Ile302Met/c.906A>G |
| chr7 | MUC3A | missense_variant | NM_005960.1:p.Thr477Ser/c.1429A>T |
| chr7 | FAM20C | frameshift_variant +stop_gained | NM_020223.3:p.Asp318_Arg319fs/c.952_953insGACAGGTGAGCCCTTCCTTC CTCCCTCCATCCGC |
|
| chr7 | MUC3A | frameshift_variant | NM_005960.1:p.Thr479fs/c.1437_1438delCA |
| chr7 | MUC3A | frameshift_variant | NM_005960.1:p.Ser481_His482fs/c.1441_1442insTG |
| chr7 | MUC3A | frameshift_variant | NM_005960.1:p.Ser1098fs/c.3292_3293delTC |
| chr7 | MUC3A | frameshift_variant | NM_005960.1:p.Arg1099_Arg1100fs/c.3296_3297insGT |
| chr9 | AQP7 | splice_donor_variant | NM_001170.1:c.407T>C |
| chr9 | PRSS3 | frameshift_variant | NM_007343.3:p.Arg158_Asp159fs/c.474_475insCC |
| chr9 | PRSS3 | frameshift_variant | NM_007343.3:p.Asp159fs/c.476_477delAC |
| chr9 | SPATA31C1 | frameshift_variant | NM_001145124.1:p.Gln75_Cys76fs/c.225_226insGC |
| chr10 | FRG2B | frameshift_variant | NM_001080998.1:p.Arg160fs/c.480delG |
| chr11 | WT1 | missense_variant | NM_024426.4:p.Ser190Cys/c.569C>G |
| chr11 | MUC6 | frameshift_variant | NM_005961.2:p.Pro1781fs/c.5341_5342delCC |
| chr11 | MUC6 | frameshift_variant | NM_005961.2:p.Thr1529_Ser1530fs/c.4585_4586insTG |
| chr11 | MUC6 | frameshift_variant | NM_005961.2:p.His1526fs/c.4577_4578delAT |
| chr11 | OR8U1 | frameshift_variant | NM_001005204.1:p.Thr53_Ser54fs/c.157_158insGA |
| chr11 | OR8U1 | frameshift_variant | NM_001005204.1:p.Ser54fs/c.161_162delGT |
| chr12 | TAS2R43 | frameshift_variant | NM_176884.2:p.Ser254_Leu255fs/c.761_762insAA |
| chr12 | GXYLT1 | stop_gained+disruptive_inframe_deletion | NM_173601.1:p.Leu223_Arg224del/c.668_670delTAC |
|
| chr12 | GXYLT1 | frameshift_variant | NM_173601.1:p.Phe222_Leu223fs/c.666_667insAA |
| chr12 | CELA1 | frameshift_variant | NM_001971.5:p.Leu4fs/c.12_15delTTAT |
| chr12 | CELA1 | frameshift_variant | NM_001971.5:p.Val3_Leu4fs/c.7_8insCT |
| chr12 | CELA1 | frameshift_variant | NM_001971.5:p.Leu2_Val3fs/c.6_7insC |
| chr12 | CNOT2 | frameshift_variant | NM_001199302.1:c.1622_1623insAA |
| chr12 | TAS2R19 | stop_lost | NM_176888.1:p.Ter300Trpext*?/c.900A>G |
| chr12 | MUC19 | missense_variant | NM_173600.2:p.Leu6611Phe/c.19831C>T |
| chr13 | PABPC3 | missense_variant | NM_030979.2:p.Glu372Gly/c.1115A>G |
| chr13 | PABPC3 | missense_variant | NM_030979.2:p.Arg374Cys/c.1120C>T |
| chr15 | HERC2 | frameshift_variant | NM_004667.5:p.Gly279fs/c.836delG |
| chr17 | SARM1 | frameshift_variant | NM_015077.3:p.Gly183fs/c.549_550delGT |
| chr17 | KRTAP4-1 | splice_acceptor_variant | NM_033060.2:c.246_302delCCCACTCTGCTGTCAGACCACCTGCCACCCCAG CTGTGGTATGTCCAGCTGCTGCCG |
|
| chr17 | EFCAB13 | frameshift_variant | NM_152347.4:p.Lys340fs/c.1018delA |
| chr17 | SPAG9 | missense_variant | NM_001130528.2:p.Val1009Met/c.3025G>A |
| chr17 | HEATR6 | missense_variant | NM_022070.4:p.Ala423Asp/c.1268C>A |
| chr19 | CD177 | frameshift_variant+stop_lost | NM_020406.3:p.Thr262fs/c.786_787delCT |
| chr19 | CD177 | frameshift_variant | NM_020406.3:p.Ser264_Cys265fs/c.791_792insAG |
| chr19 | GLTSCR1 | frameshift_variant | NM_015711.3:p.Gly1026fs/c.3077_3079delAGG |
| chr20 | SIRPB1 | frameshift_variant | NM_001135844.2:p.Leu95fs/c.284_285delTC |
| chr20 | SIRPB1 | frameshift_variant | NM_001135844.2:p.Asp94_Leu95fs/c.281_282insGT |
| chr20 | SIRPA | frameshift_variant | NM_001040022.1:p.Leu96fs/c.286_287delCT |
| chr22 | AP1B1 | missense_variant | NM_001127.3:p.Arg913Trp/c.2737C>T |
| chr22 | BPIFC | start_lost | NM_174932.2:p.Met1?/c.3G>C |
| chrX | PAGE1 | missense_variant | NM_003785.3:p.Pro42Ser/c.124C>T |
